# OA05.04. Inpatient acupuncture care: establishing hospital-wide policy and service at Beth Israel Medical Center (NYC)

**DOI:** 10.1186/1472-6882-12-S1-O20

**Published:** 2012-06-12

**Authors:** D Nielsen, B Kligler, R Lee, W Merrell

**Affiliations:** 1 Beth Israel Medical Center, Integrative Medicine Department, New York City, USA

## Purpose

Our goal was to establish inpatient acupuncture care at Beth Israel Medical Center (BIMC), a US teaching hospital that required inpatient specific policy and procedures, credentialing, supervision and a standard of safety for acupuncture therapy. Stages and logistics of ‘integrating’ modalities are identified.

## Methods

An outpatient IM clinic was established in 2000 in association with BIMC offering primary care and modalities including acupuncture therapies, which in turn established a outpatient record of safety. In 2008, we developed a post-graduate Fellowship training program in inpatient care for licensed acupuncturists, setting policy and procedures that included scope of practice, recommendations for verbal informed consent and options for individual or blanket physician permission to treat per Department. Fellows worked two four hours shifts per week for one year. The Fellowship director gave lectures on acupuncture research relevant to specialties at Department Grand Rounds. Patients verbal consent was required, an INR of 4 or less, and a platelet count of at least 25,000.

## Results

Since September 2008, Acupuncture Fellows have worked over 5000 hours with over 5,000 inpatient and staff encounters across Departments of General Surgery, Orthopedic Surgery, Family medicine, Internal Medicine, Oncology, Pulmonary Rehab, Pediatrics, and as a full consult service hospital-wide. Acupuncture therapies include acupuncture needling, ear needling, ear seeds, palpation, Tui na and Gua sha as well as explanation and recommendations in terms of traditional East Asian medicine principles. There was only one reported complication of a patient fainting that in the end was attributed to his use of his wife’s beta-blockers.

## Conclusion

It is feasible to develop a training model for graduate licensed acupuncturists as a safe and effective strategy for offering acupuncture in the inpatient setting. A safe record of care and integration of inpatient acupuncture therapy has been established at Beth Israel Medical Center in New York City.

